# Is social capital durable?: How family social bonds influence college enrollment and completion

**DOI:** 10.1371/journal.pone.0298344

**Published:** 2024-03-13

**Authors:** Mikaela J. Dufur, Toby L. Parcel, David B. Braudt, John P. Hoffmann

**Affiliations:** 1 Department of Sociology, Brigham Young University, Provo, Utah, United States of America; 2 Department of Sociology and Anthropology, North Carolina State University, Raleigh, North Carolina, United States of America; 3 Division of Behavioral and Social Research, National Institute on Aging, Bethesda, Maryland, United States of America; St John’s University, UNITED STATES

## Abstract

A large literature demonstrates that social capital has positive effects on outcomes for children, but we know little about whether social capital is durable, i.e., whether its effects persist long after its creation. We use two nationally representative data sets of U.S. high school students and structural equation modeling designed for binomial outcomes to examine the durability of returns to social capital created in the family on both college enrollment and college completion. Controlling for selected school characteristics, race, family, SES and other factors, results suggest that family social capital continues to have strong associations with outcomes increasingly distant from its creation. Family SES has a smaller but positive effect on both college enrollment and college completion. These findings suggest that social capital can be a durable good if formed in the family, and that family SES is also influential.

## Introduction

A long-standing finding in the social mobility literature points to the importance of educational attainment as intervening between social origins and adult statuses such as occupation and earnings. Thus, mechanisms to promote educational attainment are of vital importance, enabling individuals to realize upward mobility. This may be especially true of mechanisms potentially available to individuals regardless of their economic class or social status.

In this paper we analyze one such potential mechanism for facilitating such mobility. We ask, in particular, whether building social capital in adolescence can pay off in later educational accomplishments during young adulthood. We argue that social capital theory illuminates a critical pathway through which adolescents can enhance later educational attainment. In doing so, we investigate a relatively neglected aspect of social capital: whether and the degree to which its association with educational outcomes is temporally durable.

We first define social capital and identify the family as critical for its construction and use. We then argue that family social capital built during adolescence likely affects educational attainment beyond secondary school, as well as identifying both strengths and limitations of past research bearing on this issue. We next more fully develop our argument by describing potential pathways through which social capital may be durable, thus facilitating long-term educational attainment. This discussion leads to our key research question: do the effects of family social capital accrued during adolescence persist to the point of predicting college enrollment and completion? We then describe the data and methods used to address this question. The empirical model includes not only family social capital, but also relevant control variables such as school environments and family socioeconomic status (SES), both of which might confound the association between social capital and educational attainment. After describing our findings, we conclude by discussing their implications, including some limitations of our study, directions for future research, and how our findings may inform contemporary public policy.

## Adolescent social capital at home

Following Coleman [1, 2] we define social capital as involving resources that inhere in the relationships between and among actors facilitating a range of social outcomes (see Parcel et al., [3], for discussions of several types of capital). Social capital built in the family, or family social capital, refers to the bonds between parents and children promoting child socialization, which include the time and attention parents spend interacting with children, monitoring their activities, and promoting their well-being [4–7]. Similarly, we agree with Furstenberg [8], who defines social capital as a “stock of social goodwill created through shared social norms and a sense of common membership.” Families typically share norms and feelings of belonging, leading to social support and increased connections (see also [9] for a review of education-related social capital conceptualizations).

Considering children’s academic development, Coleman [1] argues that parents must invest in their children’s development and engage in interactions with them to create bonds through which norms and information can pass. These interactions begin at birth and continue through childhood and adolescence to include monitoring and enhancing children’s activities, engaging in concerted cultivation [10], and promoting all aspects of child well-being, including educational attainment (see also [11]). These investments build trust within the family, which is another aspect of social capital helpful to children’s acquisition of pro-social norms, including those that support educational attainment [12]. The focus on trust distinguishes our work from treatments of cultural capital, which focus on advantages some children may derive from parental and familial knowledge of “the rules of the game” and helps them navigate schools and related bureaucracies more successfully [13–15]. A promising characteristic of family social capital is that it can be accumulated and used even among families who are deficient in cultural or economic capital. We return to the issue of different types of capital at the end of this article.

We also rely on Putnam’s [16] distinction between bonding and bridging social capital. Intra-family connections reflect bonding social capital presumed to facilitate the positive growth of children and adolescents. But children may also benefit from the social connections that parents have with neighbors and work colleagues, or the ties parents establish when they know their children’s friends’ parents. Social connections between youth and these adults can furnish access to information about conventional norms and expectations, thus reinforcing capital built in the family. These connections represent bridging social capital to actors outside of the main family unit [17–20]. While many disciplines have used measures of such ties in attempts to predict positive child outcomes, we argue that the interactive nature of such ties, and the clear information, norms and expectations that flow across such ties, define them as social capital (cf. [19] for an example of how family social capital proves to be an efficient predictor of juvenile delinquency and how criminologists could strengthen the theoretical basis of work studying bonding by leveraging social capital theory). Bridging social capital originating in the family can be especially important when it reflects ties to actors in higher classes than the family of origin, which recent research suggests are linked to upward economic mobility [21, 22].

Coleman’s [1, 2] theoretical approach also argues that norms and expectations are forms of social capital, particularly referring to social capital shared between children and influential adults. Social capital theory, then, explains mechanisms and processes by which bonds between children and other actors, especially parents, are related to observed variation in academic attainment. Adult time and bonds with children constitute more than supervision; they create the mechanisms by which children are socialized and educated.

In this paper, we adopt this conceptualization, specifically resources that link children and parents; these resources include prosocial norms, expectations for achievement, and trust. We believe there are pathways between social capital and college enrollment and eventual completion because parents and other influential adults are likely to transmit norms and expectations concerning higher education to youth.

### Evidence for effects of social capital on adolescent educational achievement and attainment

A broad literature demonstrates that social capital promotes educational achievement, an important precursor to educational attainment. While it is true that almost all research connecting social capital and educational outcomes has been observational rather than experimental, a large and growing body of literature that uses both cross-sectional and longitudinal data and modeling suggests robust evidence for both the association between social capital and educational achievement, and that social capital created prior to the academic experience likely has a causal effect on educational achievement [3, 22, 23]. Swanson and Schneider [24] suggest that family moves during adolescence, which disrupt family social capital as well as connections to others, have negative consequences for academic achievement. On a more positive note, Parcel and Dufur [7] find that social capital in the family is associated with greater math and reading achievement for middle-grade students. Similar work demonstrates the positive effects of social capital on high school grade point average [25–28] and standardized test scores [27–31], including increases in math test scores across time [28]. These associations between family social capital and positive academic outcomes persist across students across social classes.

There is also evidence that social capital is associated with greater educational attainment. McNeal [30] finds that both parent-child discussion, a form of bonding family social capital, and parental PTA participation, reflecting bridging social capital between parents and schools, protect against school dropout [25, 32–37]. In addition, students who have higher levels of social connectedness with family members and actors outside the family are more likely to graduate from secondary schools both in the U.S. [38, 39] and in Germany [40]. Finally, Kim and Schneider [6] argue that when parental and child ambitions and educational norms align, both bonding at home and bridging to helpful sources between family members and other actors can facilitate college enrollment.

These studies underscore how children can derive important social benefits from key adults in their lives and, importantly, that the influence of such resources has been observed across a variety of domains essential for educational success. However, a continuing deficit remains in our understanding of whether the social capital created in families and other social supports provides a payoff for children’s educational attainment in the longer term.

## Durability of social capital

To be most useful in promoting educational attainment after high school graduation, family social capital would have to be durable. This is implicit in Coleman’s [1, 2] discussion of family social capital because bonds between parents and children are built and often sustained over long periods. Bourdieu’s characterization of social capital similarly emphasizes that resources derived from durable interpersonal sources are most advantageous [22, 41]. Coleman gives an explicit example of the durability of social capital when he discusses South Korean student activists who, despite having moved away from their hometowns, were connected to other activists through having come from “the same high school or hometown or church” [1]. Looking at the other end of the social capital spectrum, Coleman worries about how social capital can be lost or broken down, describing the weakening of family social capital bonds when parents divorce, which can reduce the frequency of parent-child contact or change its dynamics.

Coleman also identifies *time closure* as a resource encouraging family bonding, because when parents are committed to one another and to children over an extended time, parental investments may be greater and potentially more effective. Time closure allows family norms and expectations to persist. But social capital can decay if the parents do not continue to invest in their children across adolescence. This is analogous to financial capital, where continued monetary investments are necessary to sustain growth [42]. It also suggests that social capital built in adolescence may lose utility to influence young adult outcomes such as college enrollment and, especially, college completion, which are temporally and sometimes physically distant from the sources of that capital. Family social capital may therefore be a fungible good that is not durable and loses its utility over time.

There are conflicting arguments regarding whether social capital built during adolescence continues to pay dividends later. For some adolescents, close family ties continue after high school graduation. These ties likely receive regular maintenance during emerging adulthood if youth rely on parental advice or continued support, possibly during tertiary school enrollment [43]. But for others, high school graduation marks a time where youth begin to move away from families of origin to pursue schooling or work. In addition, peer groups become more important as children mature [44], and this shift away from family influences may become even more pronounced during early adulthood, including when youth move into tertiary educational settings. Evidence from longitudinal network analyses also suggests that major life events such as marriage and childbearing can change the nature and number of social ties young adults have [45]. We argue that transition to college attendance may be a major life course shift that affects family social capital ties and their upkeep in similar ways.

### Social capital durability and educational outcomes

Many of the studies discussed earlier measure social capital and educational outcomes simultaneously, thus precluding a study of social capital durability [4, 6, 25]. In partial contrast, looking at children pre-high school, Parcel and Dufur [7] demonstrate that family social capital has positive effects on changes in student achievement scores across two years (see also [30, 35, 39, 46] similar approaches). Although these studies ably demonstrate the durability of social capital while students are still in primary or secondary schooling, they do not test whether such capital decays as students move away from those settings in late adolescence and early adulthood.

Several researchers have investigated whether social capital, especially at home, can facilitate educational outcomes in early adulthood, but their studies have not provided definitive evidence. Furstenberg and Hughes [47] find that bonding social capital in the family as well as bridging social capital between the family and the larger community are helpful in promoting later academic and socioeconomic outcomes, effects that persist even after controlling for important socio-demographic measures. However, the small, selective nature of their sample does not allow us to generalize the findings to a broader population. Similarly, in a study of change of residence on young adult outcomes, Hagan et al. [48] discover that the negative associations between family migration and educational outcomes are more pronounced in families with uninvolved fathers and unsupportive mothers, or in other words, in families with low levels of social capital, although, again, the sample is small. More similar to our study, Ashtiani and Feliciano [49] explicitly compare the roles of social capital in three contexts affecting post-secondary attainment for low-income youth, and find that family social capital is important, although mentoring is particularly influential for low-income youth.

Studies of post-secondary enrollment are also limited. Using four waves of National Education Longitudinal Study (NELS) data, Sandefur et al. [50] determine that family social capital is a strong predictor of post-secondary enrollment, even after including rigorous controls for family financial capital, family structure, family human capital, and school social capital. However, they confine their analysis to college attendance, and thus, questions about the even more distant outcome of college completion remain (see also [51]). In addition, we argue that the distinction between college enrollment and college completion is an important one. Scholars agree that financial returns to college completion strongly outstrip returns to a high school diploma and protect against unemployment, while college degrees also confer meritocratic power [52]. However, most analyses fail to differentiate outcomes among college enrollees. Kim and Tamborini [53] argue that some non-BA terminus degrees are worthwhile, while Dadgar and Trimble [54] note variation in payoffs to lower-level credentials. Of course, many college enrollees fail to obtain any additional credential beyond their high school diplomas, thus highlighting the importance of studying what determines college enrollment and how that differs from what determines college completion.

Taken together, these studies suggest that family social capital may be consequential for late adolescent and young adult educational outcomes, particularly college enrollment. But because acquisition of a college degree has such powerful ramifications in the labor market and for later adult statuses, we also need to investigate whether the influence of social capital stretches beyond college enrollment to college completion. It may be that family social capital is strong enough to facilitate matriculation, but lacks the durability needed to influence perseverance to college graduation. The complexities of completing a college degree may outstrip the capabilities of social capital created earlier in students’ lives.

Though the possibility of durable family social capital that benefits youth throughout their lives is intriguing, we cannot be certain of the utility of this kind of capital without taking into account social contexts beyond the family. To ensure that we are accounting for these other contexts before making conclusions about family social capital durability, we also account for certain school environment factors. For example, high schools are organizations that spend considerable time teaching and modeling the norms and expectations concerning college enrollment and completion [55, 56]. Although such norms are often emphasized at school, this emphasis may vary across schools, with some having more pro-educational norms than others [5, 57]. Because of the ways primary and secondary schools function as both sorting mechanisms and training grounds for post-secondary enrollment, school environments must be considered in order to more fully test the relationship between family social capital and educational attainment. That said, we expect that school environments, no matter how important during adolescence, lose utility in promoting educational attainment as youth move on to new phases of their lives[58, 59], We wonder, then, how powerful family social capital is as youth move farther temporally, physically, and in terms of the life course from their adolescent homes, but we acknowledge the importance of taking into account school environments while exploring the potential durability of family social capital.

## Research questions

The principal research question we examine is whether family social capital is durable: does the potential influence of family social capital accrued during adolescence persist to the point of predicting college enrollment and completion? Also, do the effects of family social capital hold up in the presence of numerous controls, including school environments, family SES, secondary school academic achievement, and race/ethnicity?

## Materials and methods

### Data

Data requirements for this investigation are demanding. First, we need a national sample to allow sufficient generalizability of our findings. Second, given that we identify both families and schools as potentially relevant contexts generating social resources, the data set must contain useful measures of *both* contexts. Finally, given our interests in testing the durability of family social capital, we need longitudinal data that extend beyond students’ secondary education. Specifically, the data must include information on enrollment in and the completion of post-secondary education. We use two datasets that meet these criteria: the National Educational Longitudinal Study 1988 (NELS) [60] and the 2002 Educational Longitudinal Study (ELS) [61].

The NELS is a nationally representative study conducted by the US Department of Education and the National Center for Education Statistics (NCES) that gathered data from students, parents, teachers, and school administrators [60]. Ethics approvals for all data collection and human subjects contact were managed by NCES oversight boards, and written informed consent was provided by individuals (students over 18/teachers/school administrators/parents) or, for the minor students, parents. We use the publicly available data, which are delivered to researchers from the NCES fully anonymized. The first wave of the study was conducted in 1988, drawing random samples of approximately 25 eighth-grade students from 1,000 randomly selected schools. Because of budgetary issues, a random subsample of these students was then interviewed in 10^th^ grade (first follow-up), followed by additional interviews in 12th grade (second follow-up), two years after respondents graduated from high school (third follow-up), and eight years after respondents graduated from high school. Sample attrition, which was approximately 30.9% of the baseline sample, occurred due to the aforementioned random subsampling, refusals, drop-outs, relocations, and mortality. Attrition tended to be higher among African American and Native American students, those from the lowest SES quartile, those who scored in the lowest quartile on standardized tests, and students attending public schools. The final sample size of persistent respondents at wave 5 was 12,144. After excluding missing rows of data, we utilized a final sample size of 11,365 respondents. Our treatment of item missing data in the analysis is described later. Though we cannot be certain those who remained in the sample after the subsampling between 8^th^ grade and 10^th^ grade were similar on social capital variables and high school social characteristics measured in 12^th^ grade, which we use here, the careful nature of the NELS random subsampling method increases our confidence in the results we present below. Beyond the longitudinal design outlined above, the NELS has extensive data on schools, family circumstances, and adolescent behavior, providing a rich variety of indicators representing potential mechanisms for the accrual of social capital at home and potential measures of school environments. Another strength of the NELS is that the indicators of social capital span multiple types of actors, an important point in examining bonding and bridging social capital. [1, 2, 16]. In this study, we incorporate information on student, parent, teacher, and administrator behaviors. However, with a high school graduation date of 1990, the NELS cohort represents, relatively speaking, a cohort of students born in the early 1970s.

To address this issue, we also employ the 2002 Educational Longitudinal Study (ELS). The ELS is a nationally representative, longitudinal, study of 10th graders in the U.S. conducted by the US Department of Education and the NCES that began in 2002, with the third follow-up conducted in 2012 including variables on college enrollment and completion eight years after respondents’ high school classes graduated [61]. Ethics approvals for all data collection and human subjects contact were managed by NCES oversight boards, and written informed consent was provided by individuals (teachers/school administrators/parents) or, for the minor students, parents. We use the publicly available data, which are delivered to researchers from the NCES fully anonymized. Similar to the NELS, the ELS combines data from students, parents, teachers, and school administrators, allowing for a near-perfect match between models in the two data sets. Our sample here consists of 13,219 students who were still present in the final wave of data collection of the ELS. About 82% of eligible respondents were followed from baseline to third follow-up. Attrition was slightly higher for high school drop-outs, those from the lowest SES quartile, and those who did not attend a postsecondary institution. We acknowledge that even the ELS data were collected before the digital revolution and thus may not accurately capture all the opportunities and means youth have to build social capital today, e.g., using technology to facilitate communication, schoolwork, parental monitoring of school performance, etc. We know of no data that include such information on technology use that might facilitate the acquisition or refreshing of social capital, information on more traditional ways in which social capital is accrued through both families and schools, and longitudinal data on academic achievement, specifically post-secondary outcomes. Even with the lack of technology-based questions, the NELS and the ELS together represent a rich set of data on social capital and academic outcomes. Taken together, they also allow us to consider whether potential findings concerning the durability of social capital persist across cohorts and potential period effects, as well.

The ELS data have the advantage of being more contemporaneous than the NELS but lack some important indicators of family social capital included in the NELS, such as trust between parents and children. Using both data sets, then, takes advantage of the breadth of social capital indicators available in the NELS and the more contemporary experiences of the ELS cohort. If the results from the two datasets are similar, we gain confidence that any effects of family social capital on educational attainment are valid.

### Measures

#### College enrollment and completion

We examine both enrollment in and the completion of a college degree as outcomes (see Table 1 for summaries of all measures). Enrollment in a four-year college/university is drawn from the third follow-up wave of the NELS and the second follow-up wave of the ELS, two years after respondents’ high school graduation. Although college enrollment decisions are complicated, some research suggests that among students who delay college enrollment, most who subsequently choose to enroll do so within two years [62]. Therefore, using a variable that is two years post high school graduation allows sufficient time for most students to have made an initial enrollment decision. Additionally, prior research [52] suggests the processes involved in enrollment and graduation from two-year colleges differ from those for enrollment in four-year colleges/universities and completion of bachelor’s degrees. Consequently, we focus solely on four-year college enrollment and completion for this study.

**Table 1:** Descriptive statistics national education longitudinal study (NELS).

		Mean	sd	Min	Max
Outcomes					
Completion of 4-year degree by 2000	0 = No 1 = Yes	0.417	0.493	0.0	1.0
Enrollment in a 4-year college in 1994	0 = No 1 = Yes	0.422	0.494	0.0	1.0
Family Social Capital (12^th^ grade)					
Parents trust child	Parental report (0 = No 1 = Yes)	0.807	0.394	0.0	1.0
Discuss programs with parents	How often students report discussing school programs with parents (1 = not at all to 3 = 3 or more times)	2.318	0.654	1.0	3.0
Discuss activities with parents	How often students report discussing school activities with parents (1 = not at all to 3 = 3 or more times)	2.559	0.611	1.0	3.0
Discuss classes with parents	How often students report discussing school classes with parents (1 = not at all to 3 = 3 or more times)	2.471	0.656	1.0	3.0
Parent checks student’s homework	Parental report; 1 = never to 4 = often	3.096	0.988	1.0	4.0
Parents attend school meetings	Parental report of ever attending school meetings	0.524	0.499	0.0	1.0
Parents attend school events	Parental report 0 to of ever attending school events	0.687	0.464	0.0	1.0
Student participation in extracurricular activities	Student report; four-item scale that includes athletics<comma> academic clubs<comma> and other school extracurricular activities (α = 0.74)	22.364	2.565	19.1	57.1
Control Variables					
School Environment (12^th^ grade)					
High teacher morale	Teacher report; 1 = low to 5 = high. Higher scores = higher morale	3.971	0.902	1.0	5.0
Low conflict between teachers and administrators	Teacher report; 1 = frequent conflict to 5 = no conflict (reverse coded to reflect capital accumulation)	4.483	0.745	1.0	5.0
Teachers respond to individual needs	Parental report; 1 = never to 5 = often	4.044	0.810	1.0	5.0
School problems	14-item scale containing items asking students about the degree to which various school problems, such as delinquency, violence, and absenteeism, are a problem in their schools (α = 0.88). Coded so that higher scores = more positive environment.	33.859	4.161	11.1	40.4
Demographics					
SES (12^th^ grade)	Standardized SES scores calculated by NELS; includes both parents’ education, both parents’ occupations, family income, and a household items index.	0.017	0.758	-2.2	2.6
Female (12^th^ grade)	Respondent is Female	0.523	0.499	0.0	1.0
Family Size (12^th^ grade)	Total number of individuals living in the household	4.585	1.309	2.0	10.0
Parents Divorced/Separated (12^th^ grade)	Parental report; 0 = married 1 = divorced or separated	0.115	0.318	0.0	1.0
Race	Categorical variable from student report				
White		0.695	0.460	0.0	1
Asian		0.067	0.250	0.0	1
Hispanic/Latino		0.126	0.332	0.0	1
Black		0.092	0.288	0.0	1
Native American		0.010	0.101	0.0	1
Grades (10^th^ grade)					
English Grades	1 = Mostly below Ds; 2 = Mostly Ds; 3 = Half Cs & Half Ds; 4 = Mostly Cs; 5 = Half Bs & Half Cs; 6 = Mostly Bs; 7 = Half As & Half Bs; 8 = Mostly As	6.022	1.656	1.0	8.0
Math Grades	1 = Mostly below Ds; 2 = Mostly Ds; 3 = Half Cs & Half Ds; 4 = Mostly Cs; 5 = Half Bs & Half Cs; 6 = Mostly Bs; 7 = Half As & Half Bs; 8 = Mostly As	5.762	1.815	1.0	8.0
School discipline behaviors (10^th^ grade)					
Skipped Class	0 = Never; 1 = 1–2 times; 2 = 3–6 times; 3 = 7–9 times; 4 = O10 times	0.507	0.906	0.0	4.0
Got in trouble at school	0 = Never; 1 = 1–2 times; 2 = 3–6 times; 3 = 7–9 times; 4 = Over 10 times	0.563	0.852	0.0	4.0
In-school suspension	0 = Never; 1 = 1–2 times; 2 = 3–6 times; 3 = 7–9 times; 4 = Over 10 times	0.101	0.384	0.0	4.0
Out-of-school suspension	0 = Never; 1 = 1–2 times; 2 = 3–6 times; 3 = 7–9 times; 4 = Over 10 times	0.048	0.258	0.0	4.0
Region (12^th^ grade)					
Northeast	0 = No 1 = Yes	0.188	0.391	0.0	1.0
North Central	0 = No 1 = Yes	0.291	0.454	0.0	1.0
South	0 = No 1 = Yes	0.329	0.470	0.0	1.0
West	0 = No 1 = Yes	0.192	0.394	0.0	1.0
School Characteristics (12^th^ grade)					
Public School	0 = No 1 = Yes	0.819	0.385	0.0	1.0
Percent Lang. Minority	0 = None; 1 = Less than 10 percent; 2 = 10–19 percent; 3 = 20–29 percent; 4 = 30–39 percent; 5 = Greater or equal to 40 percent	0.744	0.956	0.0	5.0
Free/Reduced Lunch	0 = 0–10 percent; 1 = Greater than 10 percent	0.502	0.500	0.0	1.0
Educational Longitudinal Study (ELS)					
		Mean	sd	Min	Max
Outcomes					
Completion of 4-year degree by 2000	0 = No 1 = Yes	0.385	0.487	0.0	1.0
Enrollment in a 4-year college in 1994	0 = No 1 = Yes	0.750	0.433	0.0	1.0
Family Social Capital (12^th^ grade)					
Discuss programs with parents	How often students report discussing school programs with parents (1 = not at all to 3 = 3 or more times)	2.063	0.674	1.0	3.0
Discuss activities with parents	How often students report discussing school activities with parents (1 = not at all to 3 = 3 or more times)	2.119	0.717	1.0	3.0
Discuss classes with parents	How often students report discussing school classes with parents (1 = not at all to 3 = 3 or more times)	2.094	0.662	1.0	3.0
Parent checks student’s homework	Parental report; 1 = never to 4 = often	2.939	0.943	1.0	4.0
Parents attend school meetings	Parental report of ever attending school meetings	0.366	0.481	0.0	1.0
Student participation in extracurricular activities	Student report; 10-item scale that includes athletics, academic clubs, and other school extracurricular activities (α = 0.54)	1.969	1.722	0	10
Control Variables					
School Environment (12^th^ grade)					
High teacher morale	Teacher report; 1 = low to 5 = high. Higher scores = higher morale	3.801	0.833	1.0	5.0
Low conflict between teachers and administrators	Teacher report; 1 = frequent conflict to 5 = no conflict (reverse coded to reflect capital accumulation)	4.362	0.729	1.0	5.0
Teachers respond to individual needs	Parental report; 1 = never to 5 = often	3.982	0.883	1.0	5.0
School problems	19-item scale containing items asking students about the degree to which various school problems, such as delinquency, violence, and absenteeism, are a problem in their schools on a 1–4 Likert scale (α = 0.88). Coded so that higher scores = more positive environment.	69.320	7.658	35	93
Demographics					
SES (12^th^ grade)	Standardized SES scores calculated by NELS; includes both parents’ education, both parents’ occupations, family income, and a household items index.	0.036	0.750	-2.1	1.97
Female (12^th^ grade)	Respondent is Female	0.501	0.500	0.0	1.0
Family Size (12^th^ grade)	Total number of individuals living in the household	4.485	1.571	2.0	23.0
Parents Divorced/Separated (12^th^ grade)	Parental report; 0 = married 1 = divorced or separated	0.147	0.354	0.0	1.0
Race	Categorical variable from student report				
White		0.570	0.495	0.0	1
Asian		0.096	0.294	0.0	1
Hispanic/Latino		0.065	0.247	0.0	1
Black		0.133	0.339	0.0	1
Native American		0.009	0.092	0.0	1
Mixed/multiple race		0.048	0.214	0.0	1
Grades (10^th^ grade)					
Grade Point Average	Student grade point average (GPA) in 0.5 increment categories (e.g., 0 = 0–1, 1 = 1.01–1.50, 2 = 1.51–2.0, …, 6 = 3.51–4.0)	3.912	1.543	0	6.0
School discipline behaviors (10^th^ grade)					
Skipped Class	1 = Never; 2 = 1–2 times; 3 = 3–6 times; 4 = 7–9 times; 5 = 10 or more times	1.482	0.940	1.0	5.0
Got in trouble at school	1 = Never; 2 = 1–2 times; 3 = 3–6 times; 4 = 7–9 times; 5 = 10 or more times	1.639	0.917	1.0	5.0
In-school suspension	1 = Never; 2 = 1–2 times; 3 = 3–6 times; 4 = 7–9 times; 5 = 10 or more times	1.163	0.524	1.0	5.0
Out-of-school suspension	1 = Never; 2 = 1–2 times; 3 = 3–6 times; 4 = 7–9 times; 5 = 10 or more times	1.105	0.414	1.0	5.0
Region (12^th^ grade)					
Northeast	0 = No 1 = Yes	0.183	0.387	0.0	1.0
Midwest	0 = No 1 = Yes	0.249	0.432	0.0	1.0
South	0 = No 1 = Yes	0.363	0.481	0.0	1.0
West	0 = No 1 = Yes	0.205	0.404	0.0	1.0
School Characteristics (12^th^ grade)					
Public School	0 = No 1 = Yes	0.788	0.409	0.0	1.0
Percent Lang. Minority	0 = None; 1 = Less than 10 percent; 2 = 10–19 percent; 3 = 20–29 percent; 4 = 30–39 percent; 5 = Greater than or equal to 40 percent	1.016	1.142	0.0	5.0
Free/Reduced Lunch	0 = 0–10 percent; 1 = Greater than 10 percent	0.632	0.482	0.0	1.0

College completion is drawn from the fourth follow-up wave of the NELS and the third follow-up wave of the ELS, both collected eight years after respondents graduated from high school. As with college enrollment, we limit the analysis to graduation from a four-year college or university. Although the average time to completion of college degrees in the United States has increased over time, most students who complete four-year degrees do so well within the eight-year period we examine here [63, 64]. Respondents who continued their education beyond a bachelor’s degree are included in all analyses as college graduates.

#### Family social capital

We focus on family social capital that students may accrue through their bonding contact with key actors in their families and the bridging relationships those family actors have with others. The variables we use are indicative of potential capital accumulation during the student’s final year of high school. These measures span both interpersonal connections and the transmission of educational norms and are used as reflective indicators of a latent measure of family social capital.

Although we investigated multiple measurement specifications of family social capital, due to space limitations we only report results for models that use the best fitting measurement model while, as noted earlier, also taking into account social capital theory. Sensitivity analyses suggest that the inclusion of other variables theoretically linked to social capital in the measurement model did not improve model fit; consequently, they were omitted in favor of parsimony. The resulting measures of family social capital closely align with previous research that has considered structural constructs of family social capital (cf. [4, 18]).

The indicators of family social capital include avenues through which children may build social capital as they age into and through adolescence. Six of the eight indicators of family social capital are available and measured in the same way in both the NELS and ELS, while only the NELS includes indicators of parental trust and parental attendance at school events. To tap parent-child interconnectedness (bonding), we include variables on how often students discuss 1) school programs, 2) school activities, and 3) school classes with their parents, with higher scores indicating more discussion. We also include a measure of how often a parent checks their child’s homework, with higher scores indicating greater frequency of contact. Parent trust in their child (in the NELS only) is a dichotomous measure where an affirmative response indicates trust. Two items, parental attendance at parent-teacher meetings and parental attendance at school events, reflect parental interaction with schools, representing bridging social capital investments by families. Both are measured as dichotomous variables where an affirmative answer indicates having attended such meetings or events. Also indicative of bridging social capital is student participation in extracurricular activities, assessed as the sum of participation in athletics, academic clubs, and other school extracurricular activities (α = 0.74 and α = 0.54 in the NELS and ELS respectively). Participation in extracurricular activities provides more opportunity for both students and parents to build ties supportive of educational attainment. Even though the latter three variables (participation in athletics, academic clubs, and other school extracurriculars) are theoretically linked to both families and school environments, modeling the latent structure with several empirical specifications demonstrated they are more tightly linked to family social capital than to the school environment. Though most of the variables used to measure family social capital are ordinal, they all have five or more categories and are not notably skewed.

#### Controlling for school environments

School environments are also critical contexts that may support adolescent educational attainment and might account for the associations between family social capital and educational outcomes. While we treat school environments as a control variable here, the availability of multiple measures of those environments suggests a measurement model approach similar to what we describe above for family social capital. We measure school environment in secondary school with the same four items, discussed below, in both the NELS and the ELS. An indicator of student-teacher contact, which asks parents to identify the degree to which they feel teachers meet the needs of individual students, is the scaling indicator of school environment. Higher scores indicate greater connections between students and teachers. Teachers’ reports of morale and conflict between school employees provide additional indicators of school environment. High morale and low conflict are linked to greater ties to other adults in the school, providing a better overall school environment from which students can draw. We reverse-coded the original conflict measure so that higher scores on both variables would indicate greater social support. We also include a 14-item scale based on the student’s responses about their school environment, including such items as the degree to which various issues, such as delinquency, violence, and absenteeism, are a problem in their schools (α = .87 in the NELS; α = .88 in the ELS). Items in this scale are coded such that higher scores indicate more positive school environments.

#### Creating appropriate measurement models and constructs for family social capital

Understanding how family social capital is measured and whether it is even a stand-alone construct is an important part of being able to create predictive models that test our research questions concerning whether such capital is durable enough to influence postsecondary education outcomes. In previous research using the NELS data, we examined three possibilities: all youth social resources in families and schools are best represented as a single latent variable; family social capital and school environment are completely separate constructs best expressed as two latent variables; or youth social resources are best expressed as more than two latent variables, such as family social capital, global school settings, school physical resources and teacher/student ratio, and so forth. We assessed the latter two with and without allowing for cross-loadings [18]. In the current study, we tested these scenarios with the ELS data. The results suggest two distinct latent constructs, one representing family social capital and the other reflecting the quality of school environments, with no shared indicators across the two latent constructs (i.e., cross-loadings).

**Fig 1:**
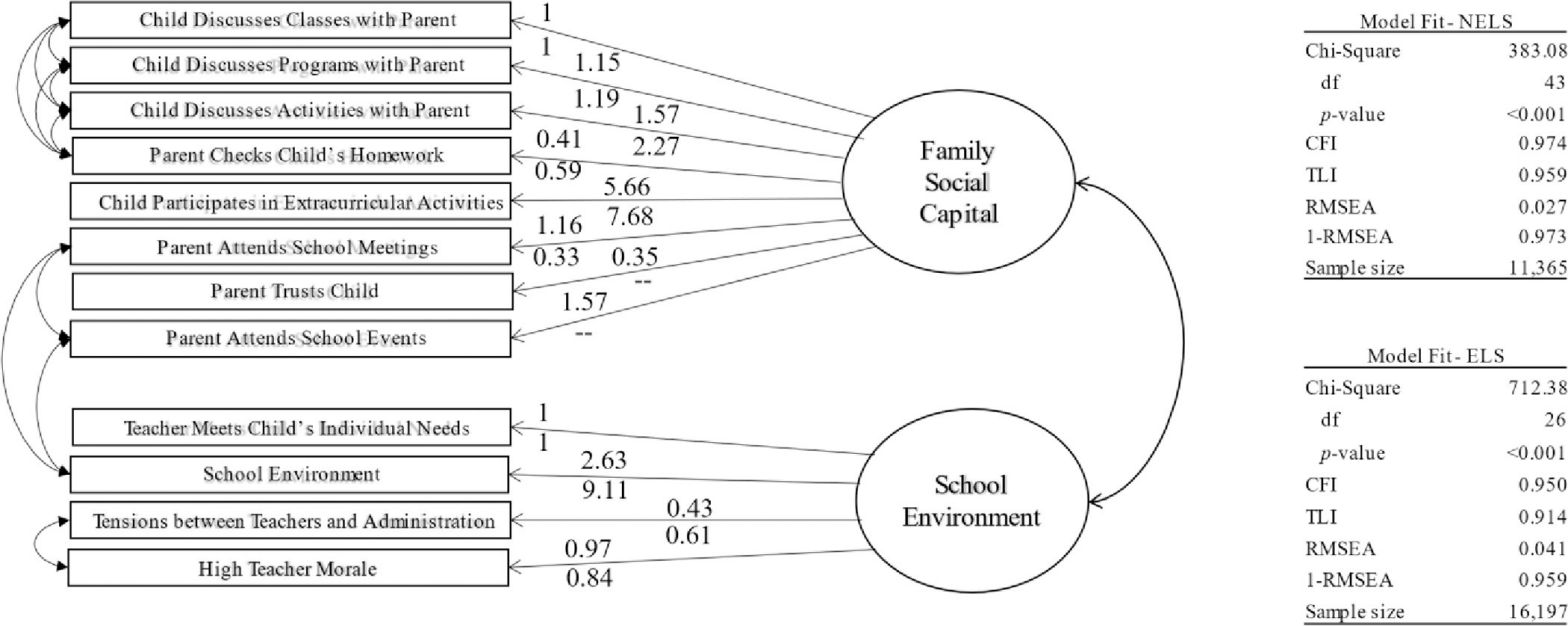
Confirmatory factor analysis of family social capital and school environments. Note: numbers above the arrows are loadings for NELS; numbers below the arrows are loading for ELS. All loadings are statistically distinct from zero at thp<0.00 I level.

The two-dimensional model presented in Fig 1 is the best fitting and most harmonious model between the two datasets. Due to similar wording in the survey questions or in some related concepts being measured, the residuals for several of the indicators for family social capital and school environments are allowed to co-vary, depicted in Fig 1 by curved two- headed arrows.

The overall model fit indices suggest that the two-dimensional model described above fits the data well, with a CFI of 0.974 (NELS) and 0.950 (ELS), a TLI of 0.959 (NELS) and 0.914 (ELS), a 1-RMSEA of 0.973 (NELS) and 0.959 (ELS), and a BIC of -8.4 (NELS) and 460.7 (ELS). For the CFI, TLI, and our transformation of the RMSEA into 1-RMSEA, a value of one represents a perfect fit. The BIC we report follows the calculation and interpretation as presented in Rafferty [65] in which a negative value indicates that the hypothesized model is a better fit to the data than a fully saturated model. The BIC for this model in the ELS data is the only overall model fit statistic that does not suggest that the measurement model fits the data well. In addition, note that the *p*-values for the chi-square statistics are small, which normally suggest that the hypothesized models do not fit the data. However, statistically significant chi-square statistics are common with large sample sizes, thus we opted to rely on the RMSEA, TLI, and CFI to guide our conclusions about model fit [66]. The correlations between the two latent constructs are 0.164 (*p* < 0.001) in the NELS and 0.266 (*p* < 0.001) in the ELS. Taken together, these measurement models leave us confident that family social capital is indeed a stand-alone construct, and that the measure of social capital we have built here allows us to proceed with longitudinal, multivariate, structural equation models that examine our research questions about postsecondary educational outcomes.

#### Additional control variables

In addition to school environment, we include several other control variables previous research suggests are strongly associated with educational attainment: parental socioeconomic status (SES) [67, 68], family size [69], student’s race/ethnicity, living with a single parent [70], and respondent’s gender [71]. To measure family SES, we use a composite scale created by NCES that includes maternal education, paternal education, maternal occupation, paternal occupation, household income, and an index of household items (see https://nces.ed.gov/statprog/handbook/els2002_keyconcepts.asp for more details). There are moderate correlations between family SES and family social capital in both the NELS (0.460) and the ELS (0.413), but we note both that these correlations suggest that SES and social capital are not overlapping inappropriately, and that previous work using similar measurement models to investigate the structure of youth’s family social capital determined that SES is an empirically distinct measure from social capital [4]. We also include control variables reflecting students’ grades, school-related behaviors, school region, and school SES. While we would prefer richer measures of school quality and school financial resources, we note that recent research found that measures of free and reduced-price lunch captured elements of educational disadvantage at the school level that measures such as aggregated and individual-level family income could not [72]. Descriptions of all these variables can be found in Table 1. Apart from high school grades and the inclusion of a multi-racial option in the ELS, all control variables are available and operationalized in the same way in both the NELS and ELS data. Including these variables provides a rigorous test of our variables of central interest: family social capital and school environments.

### Analytic strategy

We estimate a latent variable model designed to test the durability of family social capital by examining its associations with college enrollment and college completion assessed two and eight years after respondents finished high school. As such, the model includes estimates of both the direct effects of family social capital on college completion and their indirect effects, through college enrollment, on college completion. This model also includes all covariates because it fits the data better than nested models that omitted all or various subsets of covariates.

**Fig 2:**
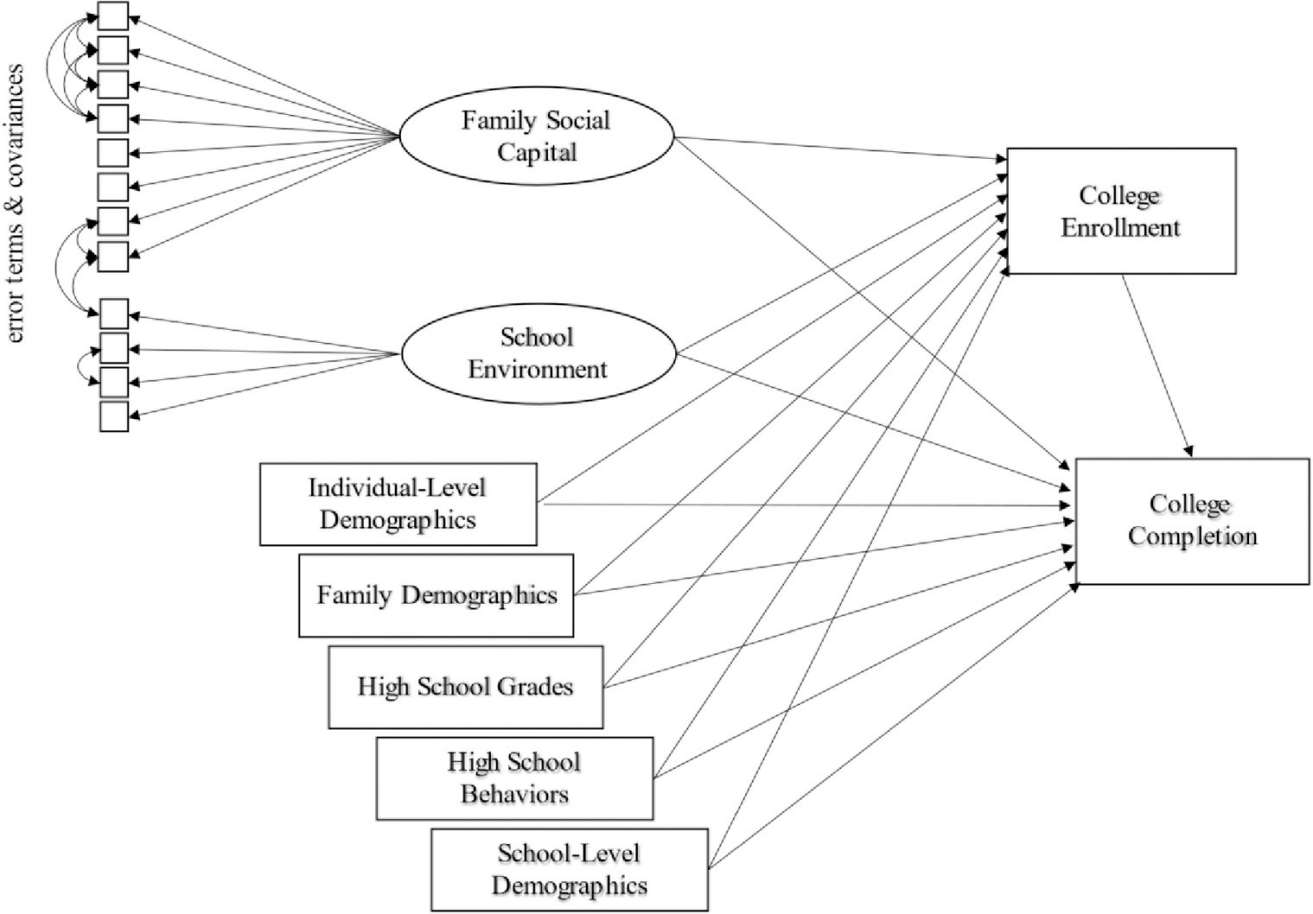
Model of college enrollment and completion with indirect and direct effects for all variables. Note: Control variable boxes in the figure are simplified for presentation. Measures represented by the "Individual-level demographics" block include: sex, race/ethnicity, and region. Measures included in "Family demographics" are: composite SES score (maternal and paternal education, maternal and paternal occupation, household income, household items index), family size, and family structure (parental marital status). Measures represented by "High school grades" include: cumulative student GPA. Measures represented by "School behaviors" include: skipping class, getting in trouble at school, in-school suspension, and out-of-school suspension. And finally, measures included in the "School-level demographics" block include whether school is public or private, percentage of students who are language minorities, and percentage of students who receive free or reduced-price lunch.

The use of categorical variables as both the mediator and outcome presents challenges to the calculation of indirect and total effects in mediation analysis. We follow Breen et al. [73] in their approach to simultaneously estimating a linear probability model and a logit model to consider our outcomes, both of which are dichotomous. The description in S1 Appendix in S1 File furnishes the analytic details of our estimation approach. While Breen et al.’s exemplar model also used college attendance and college completion as outcome variables, we note that their work was meant as a methodological exercise to demonstrate proof of their models’ ability to manage dichotomous outcomes rather than as a theoretical exploration of the pathways to educational attainment. Our work here focuses on a specific path that might be associated with greater educational attainment, the acquisition and use of social capital, as well as exploring a theoretical component of social capital (durability) while using the successful modeling approach Breen et al. describe in their work. Fig 2 provides an illustration of the full empirical model.

#### Missing data

Only about one percent of the data from the NELS and ELS were missing (item nonresponse) for the outcome variables. Less than 10% of data were missing (item nonresponse) for the other variables. We found no evidence that they were missing not at random (MNAR), so we adjusted for missing data due to item nonresponse using full information maximum likelihood (FIML) as implemented in MPlus 7.4. This ensures a constant sample size over all analyses. Research demonstrates that FIML yields results similar to multiple imputation (MI) and outperforms other methods such as random forests and stochastic regression imputation [74, 75].

## Results

Table 2 provides the untransformed results, which are used to furnish a brief illustration of how we calculated the direct, indirect, and total effects of family social capital (see S1 Appendix in S1 File, Eqs (9*)–(11*)). The OLS coefficient from the linear probability model and the log-odds from the logistic model for family social capital in the NELS data are 0.337 and 1.217 (see Table 2). The estimated log-odds for college enrollment on college completion is 2.557. Thus, the direct, indirect, and total effects of family social capital on college completion from the NELS data are as follows:

Direct Effect: Given *b*_*yx*.*z*_ = 1.217, it follows that

$$e^{1.217}=3.377$$ (1)

Indirect Effect: Given $\beta_{zx}b_{yz.x}=(0.337)(2.557)$, it follows that

$$e^{0.862}=2.367$$ (2)

Total Effect: Given $b_{yx.z}+\beta_{zx}b_{yz.x}=1.217+0.862$, it follows that

$$e^{2.079}=7.996$$ (3)

**Table 2:** Estimated linear probability model coefficients for college enrollment and log-odds for college completion, NELS, 1990–98, and ELS, 2002–12.

Explanatory variables	College enrollment OLS coefficients		College completion log-odds	
	NELS	ELS	NELS	ELS
Family social capital	0.337***	0.234***	1.217***	1.571***
School environment	-0.015	-0.048	-0.014	0.592**
Enrollment in a four-year university during the previous wave			2.557***	2.230***
SES	0.155***	0.075***	0.780***	0.519***
Female	-0.014	0.006	0.120	0.839**
Family size	-0.006	-0.016***	-0.058*	-0.058**
Parents divorced/separated	0.009	-0.012	-0.227*	-0.002
Racial/ethnic group^a^				
Asian	0.147***	0.094***	0.715***	0.574***
Latinx	0.043**	0.031*	-0.202	-0.100
Black	0.083***	0.079***	-0.193	0.109
American Indian	0.018	-0.053	-1.235**	-0.196
Multi-racial		-0.028		-0.035
GPA		0.078***		0.703***
English grades	0.045***		0.234***	
Math grades	0.034***		0.116***	
Skipped class	-0.013***	-0.004	-0.175***	-0.022
Got in trouble at school	-0.018**	0.007	-0.021	-0.033
In-school suspension	-0.016	-0.046***	-0.292**	-0.084
Out-of-school suspension	0.004	-0.048***	-0.546**	-0.253*
School-level variables				
Public school	-0.109***	-0.065***	-0.484***	-0.491***
Percent language minority	0.006	0.007	0.027	0.086*
Free/reduced lunch	-0.028**	-0.068***	-0.147*	-0.507***
Region^b^				
North Central	-0.061***	-0.029**	-0.291**	-0.603***
South	-0.079***	-0.048***	-0.291**	-0.489***
West	-0.161***	-0.035**	-0.117	-0.858***
R-square	0.335	0.303	0.627	0.656

Notes

NELS = National Educational Longitudinal Study

ELS = Educational Longitudinal Study.

The sample sizes are 11,365 for NELS and 13,250 for ELS.

^a^White is the reference category for the racial/ethnic groups.

^b^Northeast is the reference category for region.

***p<0.001

**p<0.01

*p<0.05

^+^p<0.1

Table 3 shows the results of applying Eqs (1)–(3) to the coefficients of all the explanatory variables. Enrolling in a four-year college or university has the largest direct effect on completing college (OR = 12.9 [NELS] and 9.9 [ELS]), with the odds of completion in each dataset 10–13 times higher among those who enrolled in college within two years of high school graduation.

**Table 3:** Direct, indirect, and total effects of family social capital on college completion, NELS, 1990–98, and ELS, 2002–12.

Explanatory variables	NELS, 1990–98			ELS, 2002–12		
	Direct	Indirect	Total	Direct	Indirect	Total
Family social capital	3.377***	2.367***	7.996***	4.811***	1.709***	8.224***
School environment	0.986	0.963	0.949	0.553**	0.896	0.496**
Enrollment in a four-year university during the previous wave	12.897***			9.954***		
SES composite	2.181***	1.486***	3.241***	1.680***	1.186***	1.996***
Female	1.127	0.964	1.088	0.839**	1.013	0.850**
Family size	0.944*	0.986	0.931**	0.944**	0.964***	0.909***
Parents divorced/separated	0.797*	1.023	0.816	0.998	0.971	0.969
Racial/ethnic group^a^						
Asian	2.044***	1.458***	2.980***	1.775***	1.242***	2.206***
Latinx	0.817	1.115**	0.911	0.905	1.075*	0.971
Black	0.824	1.237***	1.020	1.115	1.200***	1.338**
American Indian	0.291**	1.048	0.305**	0.882	0.886	0.782
Multi-racial	–	–	–	0.966	0.938	0.906
GPA	–	–	–	2.020***	1.196***	2.416***
English grades	1.264***	1.122***	1.418***	–	–	–
Math grades	1.123***	1.091***	1.225***	–	–	–
Skipped class	0.839***	0.967**	0.811***	0.978	0.992	0.969
Got in trouble at school	0.979	0.956**	0.936	0.968	1.016	0.984
In-school suspension	0.747**	0.960	0.717**	0.919	0.900***	0.828
Out-of-school suspension	0.579**	1.011	0.586**	0.776*	0.896***	0.694**
School-level variables						
Public school	0.616***	0.757***	0.466***	0.612***	0.861***	0.527***
Percent language minority	1.027	1.015	1.043	1.090*	1.017	1.108**
Free/reduced lunch	0.863*	0.931**	0.803**	0.602***	0.855***	0.515***
Region^b^						
Midwest	–	–	–	0.547***	0.935**	0.511***
North Central	0.748**	0.855***	0.639***	–	–	–
South	0.748**	0.818***	0.612***	0.613***	0.896***	0.548***
West	0.890	0.662***	0.590***	0.424***	0.923**	0.392***
Fit statistics						
R^2^ –College enrollment			0.335			0.303
R^2^ –College completion			0.627			0.656
Sample size			11,365			13,250

Notes

The indirect effects denote effects that are channeled through college enrollment. Exponentiated coefficients are provided. NELS = National

Educational Longitudinal Study; ELS = Educational Longitudinal Study.

^a^White is the reference category for the racial/ethnic groups.

^b^Northeast is the reference category for region.

*p<0.05

**p<0.01

***p<0.001

The results also show the durability of family social capital on the odds of completing a bachelor’s degree. Because the model adjusts for college enrollment, the direct effects denote the association of the other explanatory variables with degree completion conditional upon enrollment in a four-year college or university. Thus, adjusting for the effects of the other variables, each one-unit increase in family social capital is associated with an estimated 3.4- or 4.8-fold increase in the odds of obtaining a bachelor’s degree.

The direct effects of school environments, though, are modest and fail to attain statistical significance in the NELS data. In addition to college enrollment and family social capital, two other variables stand out for their estimated direct effect on college completion: family SES and self-identifying as Asian. Higher family SES is associated with an increase in the odds of obtaining a bachelor’s degree in both datasets, even with adjustments for college attendance, family social capital, high school grades, and several other covariates. These results are in line with those from previous research that used the example of family SES and college completion to test structural equation modeling approaches with dichotomous outcomes. Breen et al. [73], whose method we use for calculating direct, indirect, and total effects, apply the same decomposition methods to assess the association between family SES and college completion with the NELS data. They find similar direct effects (log-odds = 0.71 compared to 0.78 in our models) and total effects (odds ratio = 1.65 compared to 1.68 from our estimates) of SES. These similar findings for SES give us confidence that our models as a whole are accurate, an especially welcome finding given the strength of effects we find for our key explanatory variable, family social capital. The expected odds of college completion are twice as high among Asian students relative to White students in the NELS and about 75 percent higher in the ELS, even with adjustment for a host of other factors [76, 77].

Turning to the indirect effects, the results indicate that family social capital has a significant association with college completion via college enrollment in both datasets, thus reinforcing its role as an important durable social good. In particular, a one-unit increase in family social capital, when mediated by college enrollment, is associated with more than a two-fold increase in the odds of obtaining a bachelor’s degree in the NELS and a 1.7-fold increase in the ELS. Once again, family SES and self-identifying as Asian are also associated with a higher odds of college completion. Similarly, but to a lesser degree, self-identifying as Black and Latinx are positively associated with college completion in both datasets. Once adjustments are made for the other covariates, the odds of obtaining a bachelor’s degree, at least as routed through college attendance, are expected to be about 12 (NELS) or 8 (ELS) percent higher among Latinx and 24 (NELS) or 20 (ELS) percent higher among Black students relative to White students. This suggests a compensatory effect of attending college for some groups: even though individuals who self-identify as Black or Latinx manifest a slightly lower log-odds of obtaining a bachelor’s degree relative to White students, their expected higher odds of graduating college conditional upon college attendance within two years of graduating high school are slightly higher than those of their White peers.

The total effects represent a combination of the direct and indirect effects (see Eq 3). Supporting the view that family social capital is durable, and its influence is substantial, the results suggest that family social capital has the strongest association with whether or not one obtains a bachelor’s degree within eight years of graduating from high school (odds ratio = 7.996 [NELS] and 8.224 [ELS]). This estimated association is of larger magnitude than family SES, high school grades, and other well-established predictors of college completion. After adjusting for the other covariates, each one-unit increase in family social capital is associated with an eight-fold increase in the odds of obtaining a bachelor’s degree in both the NELS and ELS, whereas a one-unit increase in family SES is associated with about a two- or three-fold increase (odds ratio = 3.241 [NELS] and 1.996 [ELS]). This pattern persists even after adjusting for the effects of school environment, which does not seem to exert the same kind of durable effect family social capital does.

The associations of these various explanatory variables may not, strictly speaking, be directly comparable given their distinct metrics (see [78] for more detail on the measurement and scaling of latent variables), but some key comparisons are measured on a similar metric so they may be roughly compared. For instance, family SES has a range of about four or five in the datasets, whereas family social capital has a range of three, thus one-unit shifts in each imply a slightly different magnitude. But the large direct, indirect, and total effects of family social capital give us confidence that it has a powerful influence on the likelihood of graduating from college up to eight years after completing high school and is thus a durable social good. It appears, moreover, to be at least as strongly associated with college graduation as high school grades, family SES, and a few other variables shown by previous studies to be key predictors of academic success in general and college completion in particular.

## Discussion

We began by wondering whether family social capital affects college enrollment and degree completion up to eight years after high school graduation. We find that social capital constructed in and through the family during adolescence has a durable and substantial association with these two key educational milestones. This pattern persists even when taking into account important characteristics of school environments. Indeed, selected secondary school characteristics have negligible direct and indirect associations with college completion, thus suggesting that their effects are less durable than family social capital’s.

These findings corroborate previous research that has found stronger effects of family social capital on both behavioral outcomes and academic attainment compared with school resources (cf. [50]), but they also suggest that certain disadvantages in the family cannot be compensated for by the selected school characteristics we have studied here, at least in terms of college enrollment and completion. Taken together, the results provide evidence that the social ties that students have with their families during adolescence have important and durable associations with college enrollment and college completion. These ties operate both directly on college completion, and indirectly through their association with college enrollment. As noted earlier, deriving similar results across two data sets studying different cohorts of adolescents provides further validation of our findings regarding the relative durability of social capital created in the home.

In addition, our findings show that even with our rigorous controls and when including family social capital, family SES remains an important direct determinant of college completion. This finding underscores a long line of sociological research demonstrating the importance of family socioeconomic background in the life chances of adolescents and young adults. Additionally, our findings provide some insight into how race matters in college completion. In the presence of numerous controls, Asian students are more likely to graduate from college than their White counterparts. Also, Black and Latinx students have higher odds of college graduation conditional on enrolling in college within two years of high school graduation compared to Whites, despite their overall lower odds of college completion generally. These findings add nuance to our understanding of how race operates in the translation of family resources into college completion.

### Limitations and directions for future research

#### Limitations

While our findings concerning the durability of family social capital, and thus the importance of continuing to examine how social resources from families of origin help to promote mobility into at least young adulthood, are robust, our analysis does have some limitations. Owing to data limitations, we were unable to measure school social capital and were restricted to examining selected characteristics of the school environment, which limits our ability to compare different sources of social capital and their long-term durability (see also [78], regarding critiques of social capital concepts and measurement in different settings). Data sources that include measures such as student-reported relationships with teachers, degree of communication with teachers and other school personnel, and trust students have in school personnel would be helpful in accurately measuring social capital at school, an interesting analog to family social capital. It is the case that our measure of parent-reported teacher interest in students can be more easily linked conceptually to school social capital than, say, our measures of teacher morale or student-reported problems at school. Ideally, with a broader range of measures of school social capital and school environment, research could explicate the relationship between these two constructs and provide a more rigorous test of the relative effects of social capital at home and at school on both college enrollment and college completion. We also study only adolescents here; looking at the durability of family social capital generated for young children would require longitudinal data that follows these children through at least their late 20s. While we know of no such data that both follows such a long period of time and includes strong measures of social capital, children in longitudinal studies such as the UK Millennium Cohort Study and the Longitudinal Study of Australian Children are soon aging into the college-going period, potentially allowing for long-term study of social capital not available in the NELS or ELS.

Our measures of family social capital also do not consider specific parenting styles or practices, both of which are beyond the purview of our study. However, our findings tie to numerous investigations in the parenting literature that point to the supportive role that parenting can play in promoting socioeconomic attainment (e.g., [79, 80]). This suggests the need for additional research differentiating family social capital from other aspects of parenting. In addition, our findings that family social capital exerts effects at least as large as family SES should encourage investigations of social mobility that include other aspects of family life, including social capital, in addition to family SES so as to explicate how elements of the family beyond SES are operating, and how they might jointly operate.

Another avenue we were unable to examine in this study involves social network influences found outside the family environment. Some studies have shown, for example, that minority students acquire social capital from other adults in their communities—a key type of bridging social capital—and that this improves these students’ chances of educational success [22]. Moreover, we were not able to assess some important intrapersonal skills and personality traits that might differentiate those who attend or complete college versus those who do not. Traits such as motivation, persistence, and conscientiousness are related to educational success in secondary and post-secondary school [81, 82] and could stem from or be otherwise associated with family social capital or other factors present in our models.

We also recognize that there may be an upper limit to the benefits of family social capital. To the extent that family bonding translates into helicopter parenting, research has suggested that this type of parenting has deleterious effects on adolescent autonomy, competence, and appropriate motivation, which may translate into negative effects on academic attainment [83, 84], a worthy direction for future research. In addition, prior research [7] has shown that better school environments may compensate for longer maternal work hours in promoting children’s math achievement, an illustration suggesting that in situations where maternal time is more limited, school resources may be helpful, although this study focused on young children and on achievement, not attainment.

Relatedly, our investigation is unable to consider whether social characteristics such as social class (SES), race, and sex are interactive bases for the effects we have uncovered. It is possible that for specific groups of adolescents, social capital or school characteristics are more important than we have shown here. Prior research shows that girls obtain stronger returns to social capital than do boys when looking at effects on test scores [18]. In addition, it is possible that school environments are more important for attainment when family SES is lower. However, our models and findings provide a firm foundation for investigations into these important questions.

Our analyses also do not address how other components of family life contribute to the acquisition or lack of family social capital. Because family social capital has such enduring effects on attainment, our findings also highlight the need for additional research regarding the determinants of family social capital itself. For example, it is possible that stronger socioeconomic status facilitates family bonding [18], thus suggesting that family SES may have indirect effects on college enrollment and completion beyond those we have uncovered here. Similarly, the negative associations of single-parent household status and larger family size with educational outcomes suggest that these variables may have additional deleterious consequences for educational outcomes beyond the direct effects we identify in this study.

We have been unable to evaluate whether for some adolescents, family social capital might operate in a negative fashion. The overwhelming assumption in many studies is that family bonds, involvement, and trust facilitate positive educational outcomes, thus promoting social mobility [85]. But it remains possible that in some families, such bonds and involvement discourage educational attainment. For example, some parents could transmit norms of distrust in educational institutions, or norms that leaving the family to attain more education is inappropriate. Although we have uncovered no evidence for this possibility in these data, it is also true that such data to not lend themselves to understanding negative social capital. Until we know more about how the content of communications traveling through bonds and involvement may be distributed across adolescents, we will be unable to investigate this idea. It may also be that, in some cases, strong bonds with peers discourage educational attainment, thus working against pro-educational norms conveyed at home, a possibility that is beyond the scope of this research.

Sample attrition in the NELS data may also affect the results and our ability to generalize to a general population of students. As noted earlier, attrition was higher in the NELS among African American and Native American students, those from the lowest SES quartile, and students who scored in the lowest quartile on standardized tests. Though it is not clear if these factors affect social capital, they may be related to other key variables such as school environment and other school characteristics. If this does introduce bias into the models, though, the direction is unclear. Thus, future studies should assess the effects of sample attrition in longitudinal studies of family and school effects.

Finally, we have reached our conclusions only within the context of studying educational outcomes. It is important to use other longitudinal data sources to evaluate whether family social capital endures to support outcomes that occur ten to fifteen years after high school graduation, such as childbearing, non-marital fertility, or later economic attainment. These are especially important pathways to examine, not only because they are highly correlated with socioeconomic attainment and social mobility but because they are themselves important outcomes of interest for both individuals and societies [86–88]. Future research should also investigate whether social capital built in non-family contexts, such as neighborhoods or peer groups, may also be consequential, or even more durable than family social capital. For example, youth are likely to maintain enduring ties with their neighborhoods after leaving home, if only by virtue of returning to the family home. Similarly, they may be more likely to maintain close ties with friends than they are to maintain more instrumental ties—no matter how warm—with school personnel. In such cases, forms of social capital built outside the home might prove to be durable and to have long-term effects on later life outcomes. Our data were unable to address such forms of social capital, and only with a larger body of evidence regarding such associations can we come to firm conclusions regarding the conditions under which social capital, in families or elsewhere, is durable or not.

#### Implications for theory

Our findings provide important evidence regarding the durability of family social capital that support Coleman’s [1, 2] original theorizing. However, our study is limited to adolescents, and an evaluation of the durability of their social capital only built through the family. We therefore hope that our study encourages other investigators to examine whether the durability of social capital documented here persists across specific socio-demographic groups, and to explore whether that durability is also dependent on the context in which it is accrued. For example, are work or political outcomes a partial function of social capital created much earlier? Is immigrant assimilation more effective based on recent or long-standing social ties? Are families relatively unique contexts for creating the type of social capital that can have long-standing effects? Only with investigations of questions such as these can we provide the evidence needed to inform the question of social capital durability more generally, thus providing new evidence regarding social capital theory.

#### Implications for policy

To promote college enrollment and completion, it is logical that policymakers attempt to influence financial, human and social capital in schools. Schools are charged with promoting both student achievement and attainment; when one or both fall short, the school may be seen as the site requiring policy intervention. In contrast, our findings point to the importance of the family as a site for promoting college enrollment and completion. Specifically, two related avenues for intervention may be promising.

One avenue involves increasing family income and other financial resources. Consistent with a host of studies that undergird this approach, our results indicate that family SES has important direct and indirect effects on educational attainment. Higher-SES families transmit this advantage to their children through college enrollment and completion, net of any social capital captured by our measure of family norms, behaviors, and ties. Thus, programs to boost family incomes via higher wage and salary levels and opportunities for steady employment should be helpful, as should additional programs focusing on income supports such as the Earned Income Tax Credit. From a policy perspective, then, investment in family financial capital may be similar to investment in family social capital, at least in terms of encouraging college attainment.

We also find, however, that the estimated associations of family social capital are stronger than those of family SES. This suggests that while family financial means are important, they are not more important than the social capital accrued through social bonds and pro-educational norms at home. It remains possible, or even likely, that stronger family financial resources facilitate the creation of family social capital, as we noted above.

Beyond that possibility, our results suggest a second avenue for intervention. Specifically, more attention should focus on families and their capital, especially family social capital and ways that capital can be maintained, refreshed, and reinvested as children leave the family home. Policy makers should consider strengthening efforts to promote family bonding via programs that emphasize the importance of transmitting pro-educational norms to parents at all socioeconomic levels, but particularly to parents in at-risk households [89]. If successful, such programs could result in higher proportions of parents transmitting pro-educational norms to their children. Moreover, encouraging parental involvement with schools, such as attendance at school meetings and school events, can serve as important sources of bridging capital that benefit children’s educational attainment [50]. Organizational changes to workplaces, such as telework, that allow greater opportunities for family bonding may also create the kind of durable social capital that youth can access to help them achieve long-term educational attainment and thus greater socioeconomic opportunities.

## Supporting information

S1 File Structural equation modeling strategies for dichotomous college enrollment and completion outcomes. (DOCX)
