# Supplementary material for: Is social capital durable?: How family social bonds influence college enrollment and completion
Source: PLoS One. 2024 Mar 13;19(3):e0298344. doi: 10.1371/journal.pone.0298344 (PMC10936839; doi:10.1371/journal.pone.0298344)
Supplement: S1 File — (DOCX) [file pone.0298344.s001.docx]

**Supporting information**

**S1 Appendix. Structural equation modeling strategies for dichotomous college enrollment and completion outcomes**

This appendix provides details about the empirical modeling strategy designed to estimate the full models with the NELS and ELS datasets.

Beginning with the analytic decomposition of logit and linear probability models established by Breen et al. (2013), consider the two logistic models in equations (1) and (2) and linear probability models in equations (3) and (4). For simplicity, the equations below are presented in reduced form, with the variables transformed to have means of zero, thus obviating the need for an intercept term in the equations:

$logit\left( Pr\left( y=1 \right) \right)=b_{yx}x$ (A.1)

$logit\left( Pr\left( y=1 \right) \right)=b_{yx.z}x+ b_{yz.x}z$ (A.2)

$y=\beta_{yx}x+\varepsilon_{x}$ (A.3)

$y=\beta_{yx.z}x+ \beta_{yz.x}z+\varepsilon_{zx}$ (A.4)

In these equations, *x* represents social capital, *z* represents college enrollment, and *y* represents college completion.

Some researchers assume that, by estimating equations (1) and (2), they may derive an estimate of the indirect effects of *x* on *y* via *z* by examining the change in the slope (*b*) from (1) to (2), but this is not accurate. Assuming that $\varepsilon_{x}$ and $\varepsilon_{zx}$ follow a Bernoulli distribution, it follows that $\varepsilon_{x}=$ $\sigma_{e}\omega$ and that $\varepsilon_{zx}=$ $\tilde{\sigma}_{e}\varpi$ where $\omega$ and $\varpi$ are standard logistic random variables with means equal to zero and variances equal to $\frac{\pi^{2}}{3}$. From this we see that the logistic regression coefficients $b_{yx}$, $b_{yx.z}$, and $b_{yz.x}$ are merely transformations of the linear regression coefficients $\beta_{yx}$, $\beta_{yx.z}$, and $\beta_{yz.x}$ by the scaling parameters $\sigma_{e}$ and $\tilde{\sigma}_{e}$ such that:

$logit\left( Pr\left( y=1 \right) \right)=b_{yx}x=\frac{\beta_{yx}}{\sigma_{e}}x$ (A.5)

$logit\left( Pr\left( y=1 \right) \right)=b_{yx.z}x+ b_{yz.x}z= \frac{\beta_{yx.z}}{\tilde{\sigma}_{e}}x+ \frac{\beta_{yz.x}}{\tilde{\sigma}_{e}}z$ (A.6)

The $\sigma_{e}$ and $\tilde{\sigma}_{e}$ are derived from the residuals in equations (3) and (4), as noted earlier. Given the theoretical importance of both variables, we also know that $\sigma_{e}\geq\tilde{\sigma}_{e}$ since any model of $y$ that does not include $z$ as a covariate will have more unexplained variation assuming that $z$ is significantly associated with $y$. The fact that $\sigma_{e}\neq\tilde{\sigma}_{e}$ implies that any cross-model comparison of coefficients is not feasible because the scaling parameters differ between models, and the indirect and total effects in the mediation analysis which depend on these coefficients for calculation also cannot be compared [1].

The issue of these different scaling parameters between two logistic regression models confounds the interpretation of mediation models in which the mediator and outcome are both binary variables [2]. To understand why, we need to first outline the computation of indirect, direct, and total effects in mediation models in the linear case. Let $y^{*}$ and $z^{*}$ represent continuous measurements of college completion and college enrollment respectively, and $x$ represent a continuous measure of social capital. We hypothesize that $x$ is associated with $y^{*}$ both directly and also indirectly through $z^{*}$. The resulting linear mediation model is

$y^{*}=\beta_{yx.z}x+ \beta_{yz.x}z^{*}+\varepsilon_{zx}$ (A.7)

$z^{*}=\beta_{zx}x+\varepsilon_{z}$ (A.8)

The direct, indirect, and total effects are calculated as:

Direct Effect: $\beta_{yx.z}$ (A.9)

Indirect Effect: $\beta_{zx}\beta_{yz.x}$ (A.10)

Total Effect: $\beta_{yx.z}+ \beta_{zx}\beta_{yz.x}$ (A.11)

If we transform these models into logistic regression models with binary $y$ and $z$, the resulting direct, indirect, and total effects suffer from the same problem that was shown earlier: the scaling parameters will differ between the two equations in the model and confound the computation of indirect and total effects.

Breen et al. [3] present a method of estimation for mediation models with a binary outcome and a binary mediator consisting of two simultaneous equations. The first assumes a binary mediator and uses a linear probability model to estimate the relationship between an explanatory variable (*x*) and a mediator (*z*). The second uses a logistic regression model to estimate the relationships among the explanatory variable (*x*), the mediator (*z*), and the outcome (*y*). With only a single logistic scaling parameter, the indirect and total effects can be calculated in the manner prescribed by (10) and (11). To illustrate, using the logistic regression model from equation (6) and a linear probability model and substituting back in the binary $z$ in place of $z^{*}$ in equation (8), we may reconfigure equations (9) – (11) to derive calculations for the direct, indirect, and total effects:

Direct Effect: $b_{yx.z}=$ $\frac{\beta_{yx.z}}{\tilde{\sigma}_{e}}$ (A.9*)

Indirect Effect: $\beta_{zx}b_{yz.x}= \beta_{zx}\left( \frac{\beta_{yz.x}}{\tilde{\sigma}_{e}} \right)= \frac{\beta_{zx}\beta_{yz.x}}{\tilde{\sigma}_{e}}$ (A.10*)

Total Effect: $b_{yx.z}+ \beta_{zx}b_{yz.x}= \frac{\beta_{yx.z}+ \beta_{zx}\beta_{yz.x}}{\tilde{\sigma}_{e}}$ (A.11*)

As shown in the latter portion of equalities (9*) – (11*) there is only one scaling parameter $\tilde{\sigma}_{e}$, thus avoiding the issue of using coefficients from different logistic regression models. Without a loss of generality, the above computations of the direct, indirect, and total effects in the presence of a binary mediator and a binary outcome can be extended to a model with multiple covariates.

We use this approach to estimate a single latent variable mediation model as depicted in Figure 2 in the primary text. The first part uses a linear probability model to estimate the associations between family social capital, school environment, and the other covariates on college enrollment, whereas the second part uses a logistic regression model to estimate the direct effects of all covariates, including college enrollment, on college completion. Analytically, the two parts of the model may be represented as:

$z=\alpha_{z}+ \beta_{1}x_{1}+\beta_{2}x_{2}+\beta_{3}x_{3}+\boldsymbol{x}_{\boldsymbol{q}}\boldsymbol{\beta}_{\boldsymbol{q}}+\varepsilon_{z}$ (A.12)

$logit\left( Pr\left( y=1 \right) \right)=\alpha_{y}+b_{1}x_{1}+ b_{2}x_{2}+\boldsymbol{x}_{\boldsymbol{q}}\boldsymbol{b}_{\boldsymbol{q}}+b_{z}z$ (A.13)

In this specification, *y* is the binary indicator of college completion (1 = yes), *z* is the binary indicator of college enrollment (1 = yes), *x*_1_ is family social capital, *x*_2_ is school environment, and ***x_q_*** is a vector of the other covariates included in the model.

REFERENCES

1. Wooldridge JM. Introductory econometrics: A modern approach. 4th ed. Cengage Learning; 2008.

2. MacKinnon DP, Dwyer JH. Estimating mediated effects in prevention studies. Eval Rev. 1993 May;17(2):144-58.

3. Breen R, Karlson KB, Holm A. Total, direct, and indirect effects in logit and probit models. Sociol Methods Res. 2013;42(2):164-191.
